# Genetic control of anthocyanin pigmentation of potato tissues

**DOI:** 10.1186/s12863-019-0728-x

**Published:** 2019-03-18

**Authors:** Ksenia V. Strygina, Alex V. Kochetov, Elena K. Khlestkina

**Affiliations:** 1 Institute of Cytology and Genetics, Siberian Branch of the Russian Academy of Sciences, Lavrentjeva Ave. 10, Novosibirsk, 630090 Russia; 2 Novosibirsk State University, Pirogova Str., 1, Novosibirsk, 630090 Russia; 3 N.I. Vavilov All-Russian Research Institute of Plant Genetic Resources (VIR), Bolshaya Morskaya Str., 42-44, St. Petersburg, 190000 Russia

**Keywords:** Anthocyanins, Diagnostic markers, Gene transcription, *Solanum tuberosum*, Marker-assisted selection

## Abstract

**Background:**

The cultivated potato *Solanum tuberosum* L. is the fourth most important crop worldwide. Anthocyanins synthesis and accumulation in potato tissues are considered as one of important traits related to stress resistance and nutritional value. It is considered that the major regulatory gene for anthocyanin biosynthesis is R2R3 MYB-encoding gene *StAN1*. However, the genetic control of pigmentation of different potato tissues is substantially under investigated. The development of genetic markers for breeding of potato with specific pigmentation pattern remains an actual task.

**Results:**

We investigated 36 potato varieties and hybrids with different pigmentation of tubers and leaves. Sequence organization of regulatory *R2R3 MYB* (*StAN1*, *StMYBA1*, *StMYB113*), *bHLH* (*StbHLH1*, *StJAF13*) and *WD40* (*StWD40*) genes potentially controlling anthocyanin biosynthesis has been evaluated. The results demonstrated a high variability in the *StAN1* third exon and promoter region with the exception for 35 bp, containing elements for the transcription start and activation of gene expression in roots. The analysis of transcriptional activity of genes coding R2R3 MYBs, bHLHs and WD40 transcriptional factors in leaves of eight potato genotypes with different anthocyanin pigmentation was performed. The results showed a relation between the gene expression level and plant pigmentation only for the *StAN1* and *StWD40* genes, while other studied genes had either strong expression in all varieties and hybrids (*StMYBA1*, *StbHLH1* and *StJAF13*) or they were not expressed at all (*StMYB113*).

**Conclusions:**

It was found that *StAN1* is the major regulatory gene controlling potato anthocyanin synthesis. However, diagnostic markers developed for the functional *StAN1* alleles (*StAN1*^*777*^ and *StAN1*^*816*^) can not be used efficiently for prediction of potato pigmentation patterns. It is likely that the sequence organization of *StAN1* promoter is important for anthocyanin synthesis control and the development of additional diagnostic markers is necessary.

**Electronic supplementary material:**

The online version of this article (10.1186/s12863-019-0728-x) contains supplementary material, which is available to authorized users.

## Background

Potato *Solanum tuberosum* L. is an important food crop. Potato tubers contain a significant amount of polyphenols, including water-soluble pigments anthocyanins, which can occur in some genotypes [1]. Anthocyanins are widely distributed in the plant kingdom. These pigments colourize the vegetative and generative plant organs [1]. Anthocyanins presenting in fruits and flowers provide visual signals for attraction of pollinators and seed dispersers [2, 3]. Besides, anthocyanins also protect plants against various biotic and abiotic stresses due to their antioxidant properties and make possible nutritional and medicinal contribution to human health [1, 4]. It was shown that the pigmented potato genotypes (especially with red and purple skin and flesh) have significantly higher antioxidant activity [5]. Thus, potatoes with a high content of anthocyanins are important in terms of health. From the other side, colouration of the stem and leaves as adaptive features are noteworthy [6].

**Fig. 1:**
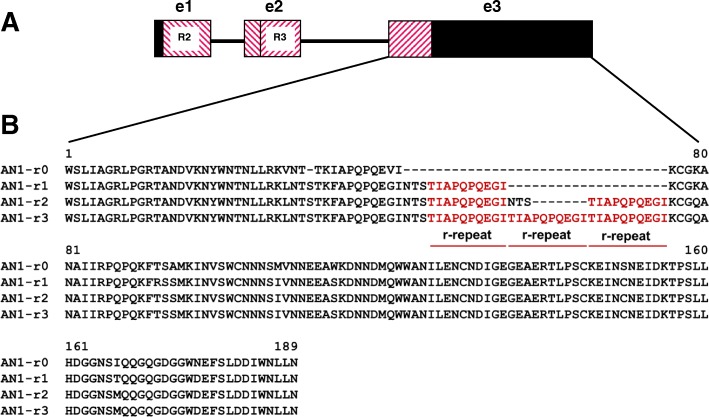
The features of *StAN1* organization. **a** Exon-intronic organization. Exons are indicated by rectangles. DNA-binding R2 and R3 MYB domains are indicated with diagonal lines **b** Protein sequences of the third exon of different *StAN1* alleles; r-repeats are highlighted below

A number of genes described previously regulate the biosynthesis of anthocyanins. It is considered that the major regulators are the genes encoding the transcription factors R2R3 MYB, bHLH (basic helix-loop-helix) and WD40 forming the MBW complex (MYB-bHLH-WD40) [7–9]. There were several R2R3 MYB-encoding genes considered to be the regulators of phenylpropanoid biosynthesis pathway, among them *StAN1* (*ANTHOCYANIN1*) was the main candidate gene for regulation synthesis of anthocyanins in potato tubers and leaves [10–13]. It has been suggested that the presence of one or two r-repeats (perfect 30 bp long duplication coding for 10 amino acids TIAPQPQEGI; alleles *StAN1-r1* and *StAN1-r2*, respectively) in the third exon of the *StAN1* gene is crucial for its proper regulatory functioning (Fig. 1) [13, 14]. Besides, the expression of *StAN1* correlated with the level of flavonoids in drought-stressed potatoes [15]. Expression of the highly homologous gene *StMYBA1* / *StAN2* was noted in tubers of pigmented and uncoloured genotypes [13]. Functional studies have shown that *StMYBA1* is less able to induce anthocyanins than *StAN1*, but it has a strong ability to induce production of monolignol and hydroxycinnamic acid derivatives [16, 17]. It has been shown that in a cold-resistant wild potato *S. commersonii* this gene is capable of responding to cold stress [16]. The R2R3 MYB-encoding gene *StMYB113* (homologous to the *Arabidopsis* gene *AtMYB113*, which positively regulates the metabolism of phenylpropanoids) also demonstrated transcriptional activity in potato tubers [13]. The bHLH-encoding genes *StbHLH1* and *StJAF13* show a correlation with the expression of phenylpropanoid genes in potato leaves and tubers [11–13]. It has been revealed that *StJAF13* regulates the expression of *StAN1*, which then activates the structural genes *CHI*, *F3H*, *F3’H* and *ANS* of tobacco plant [18]. In addition, it has been shown that the expression level of WD40-coding gene *StWD40* correlates with total phenolics and anthocyanins content in the red and purple potato tubers [11]. Further investigation of the regulatory genes related to anthocyanin biosynthesis in potato, and development of DNA markers diagnostic for their dominant and recessive alleles would provide the base for the accelerated breeding of potato with desired skin and flash colour.

Current study is aimed on (i) characterization of the *StAN1* gene and development of DNA markers diagnostic for the functional alleles of this gene as well as (ii) investigation of the functional allelic variability of the known R2R3 MYB-, bHLH- and WD40-coding genes among eight potato genotypes differing in anthocyanin pigmentation.

## Results

### *StAN1* allelic variability

#### Third exon

**Fig. 2:**
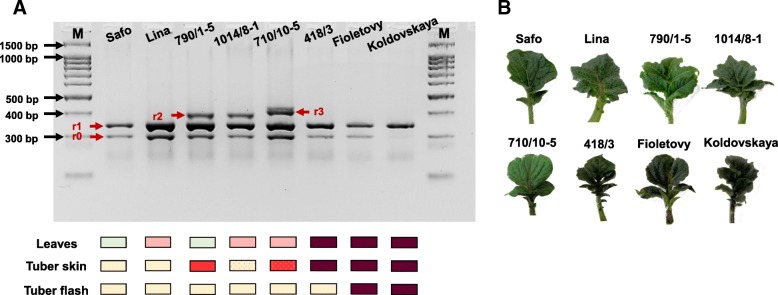
**a** PCR analysis of selected potato samples. Rectangles marked colour of leaves and stems, tuber skin and tuber flesh of potatoes. r0 – *StAN1-r0* (296 bp), r1 – *StAN1-r1* (338 bp), r2 – *StAN1-r2* (377 bp), r3 – *StAN1-r3* (398 bp). **b** Phenotype of leaves and stems in selected potato samples

High variability in the *StAN1* genomic sequence was previously identified in the 3rd exon [12–14]. The ability to regulate the anthocyanins biosynthesis in potato is provided by the presence in *StAN1* of one or two perfect r-repeats (Fig. 1). We developed DNA marker based on *StAN1* alleles inclusive from zero to three repeats (Table 1). This marker flanks all r-repeats in the third exon of *StAN1* gene. Thus, it is possible to select the functional alleles of *StAN1* by the amplicon length: *StAN1-r0–*296 bp, *StAN1-r1–*338 bp, *StAN1-r2–*377 bp, *StAN1-r3–*398 bp. The marker suitability was confirmed by sequencing of the corresponding PCR products. With these markers, 36 DNA samples of potato varieties and hybrids with different anthocyanins pigmentation of tubers skin (no/pink/red/purple), tuber flash (no/purple) and leaves (no/purple) were analysed (Table 2, Fig. 2a, Additional file 1). Functional alleles *StAN1-r1* and *StAN1-r2* were identified in 36 and 18 samples, respectively. All samples with the exception of Zlatka and 1013/3–1 have the non-functional allele *StAN1-r0*. In five samples, *StAN1-r3* allele was found (Aroza, Favorit, 419/8–1, 710/10–5, 478). Thus, it was established that all the potato samples hold a functional *StAN1* allele for anthocyanin biosynthesis activation.

**Table 1:** Gene-specific primers used in current study

Gene	GenBank ID	Purpose	Annealing temperature (°C)	Forward primer (FP, 5′ → 3′)	Reverse primer (RP, 5′ → 3′)	Primer binding site	
						FP	RP
*StAN1*	KM822778 AY841130 AY841128 KM822780	diagnostic PCR, sequencing	55	GGAAGGACAGCTAACGATG	AGTGTTCTTTCAGCTTCTCC	E3	E3
	chr10:51745200–51,749,200	PCR, sequencing	50	GTCACATCACTACACCACAT	TCCACTTCATCCCAATCAAAG	promoter	E2
	AY841128	full length CDS amplification, sequencing	50	ATGACTTCACATGTAATGATCAT	CTAATTAAGTAGATTCCATATATCA	E1	E3
	AY841128	qRT-PCR (a)	60	GGAGAAGGAAAGTGGCATCTTGTTCCA	TCCACTTCATCCCAATCAAAG	E1	E2
	AY841128	qRT-PCR (b)	60	GAGAAGCTGAAAGAACACTACCT	CACCATCACCTTGTCCTTGT	E3	E3
*StMYBA1*	JQ219855	qRT-PCR	60	GTGGTCACTTATTGCTGGTAGA	GGCGAGGAGGAGGAGTAATA	E3	E3
*StMYB113*	KU242748	qRT-PCR	60	CTAGGCAACAGATGGTCACTTAT	CTTCCTGTGTAGGTGTGTGTT	E3	E3
*StbHLH1*	JX848660	qRT-PCR	60	CCACCAAAGCCAGCTTTATC	ATCCGCTGGACAAATACCAG	E5	E5
*StJAF13*	KP317176	qRT-PCR	60	CTGCAGAGCAGACATCTGATAA	GCAGCTTTCAGGTTCCATTTC	E7	E7
*StWD40*	JX848661	qRT-PCR	60	ACCCTTAAGCCTGTTCCAAATC	CACCGGAAGAGGCAAGAATATC	E1	E1

**Table 2:** Genetic stocks of potato samples and their phenotypic characteristics that were used to characterize *StAN1* alleles. Cultivars and hybrids used for qPCR amplification are underlined

№	Cultivars and hybrids designation	Presence of leaf and stem anthocyanin coloration	Tuber coloration		Pedigree of the hybrids	*StAN1* alleles
			Tuber flesh	Tuber skin		
1	Zlatka	–	yellow	yellow with redbuds		*r1, r2*
2	Yuna	–	yellow	pink		*r0, r1*
3	Safo	–	white	white		*r0, r1*
4	Lina	+	yellow	yellow		*r0, r1*
5	710/10–5	+	yellow	red with whitebuds	Symphonia x Adretta	*r0, r1, r3*
6	1–7-5A	–	yellow	red	Zhukovsky ranniy x Ute	*r0, r1, r2*
7	1–9-2	–	white	white	1409–4/86 x Rossiyanka	*r0, r1*
8	2–5-4B	–	yellow	red	244–1 x Karlena	*r0, r1, r2*
9	1–14-2A	–	yellow	white with pinkbuds	Helena × 946–3	*r0, r1, r2*
10	1014/3–1	–	white	white with pinkbuds	Nikulinsky x Picasso	*r0, r1, r2*
11	821/1–5	–	yellow	white with pinkbuds	Nikulinsky x Picasso	*r0, r1, r2*
12	419/8–1	–	yellow	pink	Zhukovsky ranniy x CM №1	*r0, r1, r3*
13	1014/8–1	+	cream	white with pinkbuds	Nikulinsky x Picasso	*r0, r1, r2*
14	1013/3–1	–	white	white with pinkbuds	Nikulinsky x Picasso	*r0, r1*
15	790/1–5	–	yellow	red	Nikulinsky x Omega	*r0, r1, r2*
16	597/4–1	–	yellow	red	Zhukovsky ranniy x CM №1	*r0, r1, r2*
17	999/1–1	–	cream	white	Sentyabr x Latona	*r0, r1, r2*
18	785/8–5	–	yellow	red	Symphonia x Pushkinets	*r0, r1, r2*
19	2–5-2	–	yellow	yellow	244–1 x Karlena	*r0, r1*
20	826/1–5	–	white	white with pink buds	Granola × 234–90	*r0, r1, r2*
21	Favorit	+	cream	pink		*r0, r1, r3*
22	Liubava	+	white	red		*r0, r1*
23	Fioletovy	+	purple	purple		*r0, r1*
24	Koldovskaya	+	purple	purple		*r0, r1*
25	Nakra	+	yellow	red		*r0, r1, r2*
26	Meteor	+	yellow	yellow		*r0, r1*
27	Kuznechanka	+	cream	red		*r0, r1, r2*
28	Aroza	+	yellow	red		*r0, r1, r3*
29	Fritella	+	white	white		*r0, r1*
30	418/3	+	white and red	purple	Picasso x Adretta	*r0, r1*
31	479/5	+	yellow	yellow	Ausonia x Naiada	*r0, r1*
32	417/2	+	yellow	red	1–11-5 x Pamyati Osipovoy	*r0, r1, r2*
33	Bekas	+	white	white		*r0, r1*
34	Fregata	+	white	white		*r0, r1, r2*
35	478	+	yellow	red	Irbitsky x Naiada	*r0, r1, r3*
36	1753/6	+	yellow	purple	BP 808 × 88.34 / 14	*r0, r1*

#### Promoter region and 1st intron

**Fig. 3:**
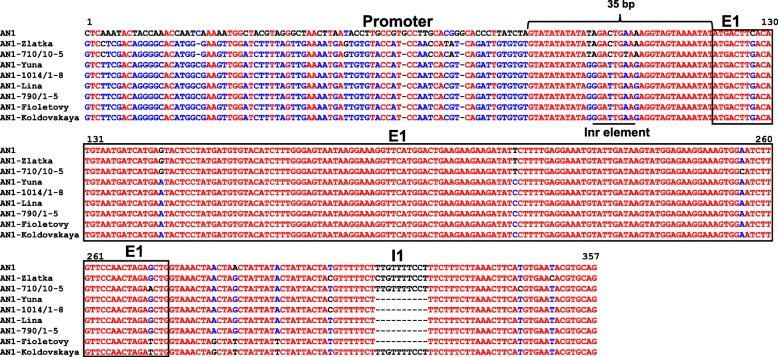
Section of a sequence alignment of *StAN1* promoter, first exon and first intron structure. High consensus colour is red, low consensus colour is blue, neutral colour is black. The location of putative transcriptional Inr element is highlighted below

Low conservatism was previously noted for the *StAN1* gene including the promoter region. In addition to the promoter, the intron 1 often acts as a regulator of gene transcription [19, 20]. Therefore, mutations in these regions could be critical in the transcription regulation. *StAN1* promoter and 1st intron sequences of eight randomly selected potato samples and one sequence from PGSC database (chr10:51745200,51,749,200) were studied here. The 15 bp indel mutation in the 1st intron was found both in pigmented and unpigmented genotypes (Fig. 3). Minor variation between individual promoter sequences of potato samples were found including single nucleotide deletion and polymorphisms. However, the analysed potato samples were similar to each other in the promoter region and completely different from the database sequence except for 35 bp (Fig. 3). The analysis with the New PLACE database revealed that the conservative region of *StAN1* promoter sequences share light-responsive Inr (initiator) element with signal sequence YTCANTYY which is necessary for promoters without TATA boxes (Fig. 3, Additional file 2). In addition, all promoter sequences have a set of common elements like root-specific elements CTCTT and ATATT, tetranucleotide CACT of mesophyll expression module and CAAT-boxes (promoter consensus sequence) (Additional file 2).

### Expression analysis of regulatory *R2R3 MYB*, *bHLH* and *WD40 *genes

Among 36 potato varieties and hybrids, eight samples characterized by marked colour differences of leaves, tuber flesh and skin were selected: Safo, Lina, Fioletovy, Koldovskaya, 710/10–5, 1014/9–1, 790/1–5, 418/3 (Fig. 2b, Table 2). From the leaves of these plants, RNA preparations were isolated to assess the expression levels of the *R2R3 MYB*, *bHLH* and *WD40* genes involved in anthocyanin biosynthesis control.

#### Analysis of *StAN1*, *StMYBA1* and *StMYB113* in differentially pigmented potato leaves

**Fig. 4:**
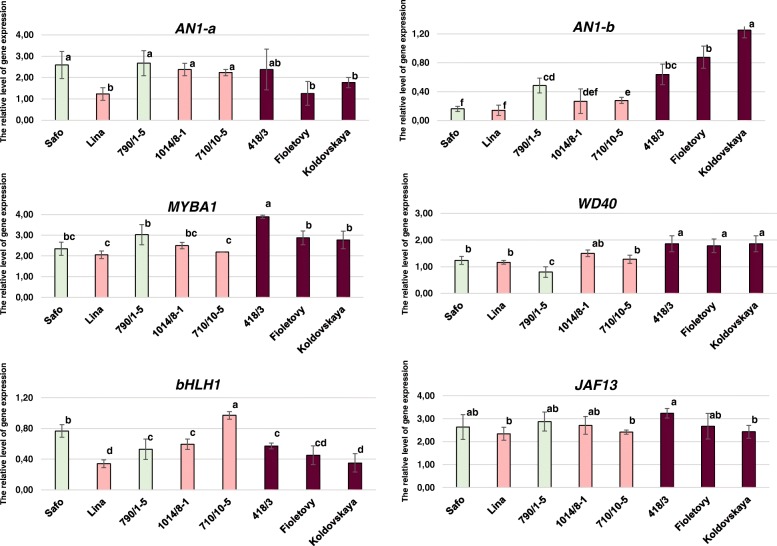
The expression of the *MYBs*, *bHLHs* and *WD40* genes in potato leaves having different colouration. The colour of the column corresponds to the colour of leaf. The data represent the means ± standard error of three biological replicates. Statistical significance was determined by one-way analysis of variance. Significant differences between means are indicated where letters above the bars differ (*p* ≤ 0.05)

To investigate the expression profiles of *R2R3 MYB* gene *StAN1* in potato leaves, two primer pairs were designed for different gene regions (Table 1, Additional file 3). It was found that different primer pairs (**a** and **b**), used in qPCR analysis, give different expression results (Fig. 4). *StAN1* expression in the R2R3 domain region (primers combination **a**) was higher in genotypes without or with weak leaf anthocyanin pigmentation than in coloured genotypes Koldovskaya and Fioletovy (Fig. 4). On the other hand, the expression of this gene in the third exon region (primers combination **b**) is associated with leaves pigmentation. We assume that the second primer pair (**b**) was more specific to detect functional *StAN1* alleles. An exception was genotype 790/1–5, in which we detected high level of *StAN1* expression despite the absence of leave anthocyanin pigmentation (Fig. 4). Full-length coding sequence of only *StAN1-r2* allelic variant was amplified from cDNA of leaves of 790/1–5 hybrid. For comparison, only one *StAN1* allelic variant named *StAN1-r1* was also amplified for genotype Fioletovy with strong anthocyanin pigmentation (GenBank: MH796171-MH796172).

The relative expression levels of the R2R3 MYB-coding genes *StMYBA1* and *StMYB113* were also analysed*.* We examined the transcriptional activity of *StMYB113*. Its mRNA was not detected in the leaves of any genotype. Expression of *StMYBA1* was detected at a high level in the analysed tissue (Fig. 4), but there was no relationship between relative level of gene expression and the presence of anthocyanins in potato leaves.

#### qPCR analysis of *StbHLH1*, *StJAF13* and *StWD40* in differentially pigmented potato leaves

Expression levels of two *bHLH *(*StbHLH1* and *StJAF13*) and one *WD40* (*StWD40*) transcription factor genes was studied. The transcription of *StbHLH1* was detected all analysed genotypes (Fig. 4). However, the strongest expression level was detected in uncoloured cultivar Safo and in hybrid 710/10–5 with weak leave pigmentation. *StJAF13* gene demonstrated the transcriptional activity at approximately the same high level in all genotypes, regardless of their pigmentation (Fig. 4). From the other side, it was shown that the strong expression level of the *StWD40* gene was associated with the anthocyanin colouration: in genotypes 418/3, Koldovskaya and Fioletovy with strong anthocyanin coloration relative expression level was significantly higher than in unpigmented genotypes Safo and 790/1–5 (Fig. 4).

## Discussion

Plant pigments anthocyanins belong to a large group of plant phenolic compounds called flavonoids. Many studies have shown health benefits with a significant amount of regularly consumed anthocyanins, which potentially helps in preventing various chronic pathologies [1]. However, most plants contain only small amounts of anthocyanins in edible parts [4, 21]. Epidermis coloured with anthocyanins and uncoloured flesh, consisting for example of white parenchymal cells, is the most common pigmentation of plant organs. This pigmentation occurs in purple cabbage, tomatoes, onions, eggplants and grapes. Nevertheless, there are examples of plants with edible organs with parenchyma coloured with anthocyanins, such as *S. tuberosum* with purple flesh [22].

The anthocyanin biosynthetic genes are transcriptionally regulated by ternary MBW protein complex containing R2R3 MYB, bHLH and WD40 transcription factors [7–9]. To date, there are three R2R3 MYB-encoding genes potentially involved in the biosynthesis of anthocyanins in the potato genome: *StAN1*, *StMYBA1* and *StMYB113*. Additionally, two bHLH genes (*StJAF13* and *StbHLH1*) and one WD40 (*StWD40*) gene have been revealed in *S. tuberosum* genome. The genes *StAN1*, *StbHLH1* and *StJAF13* have been identified as positive regulators for pigmentation of potato leaves and tuber skin and flesh [12, 13]. In contrast to conservative bHLH- and WD40-encoding genes, the MYB-encoding *StAN1* gene is variable [14].

### Major variants of *StAN1* present in potato cultivars and hybrids

Previously, it was shown that for the proper functioning *StAN1* should contain one or two perfect r-repeats in its 3rd exon (Fig. 1). These alleles were designated as *StAN1-r1* and *StAN1-r2*, respectively (formerly *StAN1*^*777*^ and *StAN1*^*816*^ [10]). It was found that in all potato samples that we analysed the functional *StAN1-r1* and *StAN1-r2* alleles are contained (Fig. 2a, Additional file 1).

Although the structure of the 3rd exon of the gene plays the key role in the anthocyanins biosynthesis [14], it appeared to be not the only one critical element – the differences between allelic variants should also be found in the promoter region. However, our results demonstrated a high variability in the *StAN1* promoter with the exception of 35 bp, containing elements of the transcription start and activation of gene expression in the plant roots (Fig. 3, Additional file 2). Apparently, some certain *cis*-regulatory enhancer element can determine the activity of the gene. Perhaps this is a putative SINE (short interspersed elements, (TA)_36_ repeat at − 1969 bp from the ATG site) element detected in D’Amelia et al. [12]. The presence of a putative SINE retrotransposon in *StAN1* promoter may explain differences between green leaf samples and leaves with abundant anthocyanin pigmentation. A similar pattern was observed for the bHLH-like transcription factor *TaMyc-A1* in *Triticum aestivum* genome, which is involved in the anthocyanins biosynthesis in wheat pericarp [23, 24]. The difference between *TaMyc-A1* alleles are in variation of number of the 261 bp-element upstream the transcription start site. Tandem duplication of this element has influence on activation of *TaMyc-A1* expression and appearance of abundant anthocyanin coloration in wheat pericarp. Thus, for the selection of potato varieties with anthocyanin pigmentation it is necessary to develop additional PCR markers flanking putative SINE element.

### *StAN1 *and *StWD40* regulate anthocyanins synthesis in leaf

It was detected that within the structural part of the *StAN1* gene, the first exon is the most conserved region (Fig. 3). To analyse the relative expression level of the *StAN1* gene, we developed primers to this region containing necessary R2R3 motif (combination **a**) and to the 3rd exon with perfect r-repeats (combination **b**) (Table 1, Additional file 3). Our analysis revealed that the primer pair **b** was proved to be more suitable to detect functional *StAN1* alleles (Fig. 4). A similar difference has been observed in Liu et al. [13] in the analysis of *StAN1* expression in potato tubers. It was found that the truncated version of *StAN1-r0* (*StAN1-r0T*) was amplified from cDNA of white skinned potato sample at positions 1–302 bp. The truncated version of *StAN1* did not promote anthocyanin synthesis at any level or significantly inhibit the activity of full-length *StAN1*. Thus, we assumed that major transcription variants of *StAN1* amplified from cDNA of uncoloured or weakly pigmented genotypes are truncated.

The independent colour of potato leaves and tubers is determined by the functional MBW complex, wherein the MYB component is the product of *StAN1* gene, but bHLH and WD40 components could be different [13, 14]*.* Indeed, a comparative analysis of *StAN1* expression with primers combination **b** revealed a relationship between the intensity of the anthocyanin colour of the plant and the level of its expression (Fig. 4). Among the samples analysed *StAN1* expression was not associated with anthocyanin pigment only in 790/1–5 hybrid sample. Due to the presence of red anthocyanin coloration in skin of hybrid tuber, we assumed that the lack of pigments in the leaves is related to the mutations in a co-regulator gene (presumably bHLH), controlling pigmentation in the leaves, but not in the tubers.

The relationship between the transcription level and the phenotype of the potato was also observed for the *StWD40* gene. Despite the fact that the level of its expression was high in plants of all the potato genotypes tested, the tendency of increased expression in pigmented samples and decreased expression in green ones was noted (Fig. 4). Expression patterns of *StWD40* suggest that it is an important factor determining anthocyanins amount in potato leaf. Previously, the expression of this gene was not detected in leaves, but a similar pattern was observed in potato tubers.

The relationship between the expression of other regulatory genes and the phenotype of the analysed tissue was not revealed (Fig. 4). It was established that *StMYBA1* is expressed in all analysed genotypes. Previously it was shown that in a cold-resistant wild potato *S. commersonii* an ortholog of this gene activated production of monolignol and hydroxycinnamic acid derivatives, which probably may be related with cold tolerance [16]. It is possible that in *S. tuberosum* the function of *StMYBA1* was lost during the evolution and domestication. *StMYB113* demonstrated complete absence of expression in the leaves of the potato. Previous study demonstrated the presence of *StMYB113* transcripts in potato tubers regardless of their pigmentation [13]. Thus, the gene *StMYB113* has a tissue-specific expression.

Previously, the relative levels of expression of both bHLH genes (*StJAF13* and *StbHLH1*) in the leaves were measured only in the paper of D’Amelia and co-authors: *StbHLH1* transcription showed no association with colour, while for *StJAF13* association was found [12]. In our research expression of *StJAF13* and *StbHLH1* in Russian potato samples was not associated with the leaf phenotype. However, we demonstrated that *StJAF13* has a conservative expression profile, even in the absence of pigmentation. These data revealed that the most important regulator controlling potato anthocyanin synthesis is *StAN1*. This gene should be targeted for further marker-assisted selection.

## Conclusions

It was found that anthocyanin synthesis in potato leaves is mainly controlled by regulatory *StAN1* gene. The structural organization of *StAN1* gene in different potato cultivars was determined. The intragenic diagnostic marker for detection of different *StAN1* alleles according to the number of r-motifs was developed. For the first time it was demonstrated that the expression of another transcription factor *StWD40* is also related to the colour of the potato leaves. These results provided new information on genetic control of potato pigmentation and may also be useful for further development of diagnostic marker for potato breeding.

## Methods

### Plant materials

Plant material were selected from GenAgro genetic collection and includes 36 varieties and hybrids of the Russian tetraploid potato *S. tuberosum* (Table 2). These potato samples were screened for the presence of the *StAN1* allelic variants (Table 2). Eight samples contrasting in anthocyanin pigmentation were selected for analysis of the expression of anthocyanin biosynthesis genes (Table 2, grey colour). The plants were grown in greenhouse facility of Institute of Cytology and Genetics SB RAS under a 14 h photoperiod.

### DNA and RNA extraction, cDNA synthesis

Total genomic DNA was extracted from leaf material applying a DNeasy Plant Mini Kit (QIAGEN, Germany). For total RNA extraction from fresh potato leaves a Plant RNA Mini Prep™ kit (Zymo Research Corporation, USA) was used. Three biological replicates were prepared for each genotype. All isolated RNAs were treated with RNase-free DNase set (QIAGEN, Germany). Total RNA was converted to single-stranded cDNA from a template consisting of 0.8 μg of total RNA using a RevertAid First Strand cDNA Synthesis Kit (Thermo Fisher Scientific Inc., USA). Testing of the effect of DNase for DNA digestive was verified by subsequent PCR with primers to the reference *Actin* gene (GenBank: X55749) flanking the intron (5’GATGCTCCACGAGCTGTATT3’ / 5’TTCACGTCCCTGACGATTTC3’).

### In silico analysis and primers design

Multiple sequence alignment was carried out using MultAlin (https://multalin.toulouse.inra.fr/multalin). Promoter analysis was made with New PLACE database (https://sogo.dna.affrc.go.jp/cgi-bin/sogo.cgi?lang=en&pj=640&action=page&page=newplace). Diagnostic primers for the determination of different *StAN1* alleles, as well as primers for the amplification of *StAN1*, *StMYBA1*, *StMYB113*, *StbHLH1*, *StJAF13* and *StWD40* transcripts, were designed using the OLIGO software. Sequences of primers, as well as conditions for PCR, are shown in Table 1.

### PCR, sequencing

Amplification was made in 20 μL PCRs according to [23]. The PCR products were separated in an agarose gel (Medigen, Russia; HydraGene Co., China), coloured with ethidium bromide. We have used and utilized gels stained with ethidium bromide according safety instructions. The gel image was obtained using a Molecular Imager Gel Doc XR System (Bio-Rad Laboratories, USA) using UV light. Isolation of the PCR products from the agarose gel was performed by the QIAquick Gel Extraction Kit (QIAGEN, Germany). DNA sequencing was carried out using the SB RAS Genomics core facilities (Novosibirsk, Russia).

### Quantitative real-time PCR (qRT-PCR)

qRT-PCR was performed with the primers from Table 1. The amplifications were performed in an ABI Prism 7000 Sequence Detection System (Applied Biosystems, USA). The subsequent qRT-PCR was based on a SYNTOL SYBR Green I kit (Syntol, Russia). The reference sequences used were 18S rRNA (GenBank: X67238) and b-tubulin (GenBank: 609267) (primers 5’GTGACGGGTGACGGAGAAT3’ / 5’ATTTATTGTCACTACCTCCCCG3’ and 5’AGCTTCTGGTGGACGTTATG3’ / 5’ACCAAGTTATCAGGACGGAAGA3’, respectively). Each sample was run in three technical replications. Statistical significance was determined by Kruskal-Wallis test with Statistica (http://statsoft.ru/). Significant differences between means are indicated where letters above the bars differ (*p* ≤ 0.05).

## Additional files

Additional file 1: PCR analysis of 36 samples of Russian potato varieties and hybrids. r0 – *StAN1-r0* (296 bp), r1 – *StAN1-r1* (338 bp), r2 – *StAN1-r2* (377 bp), r3 – *StAN1-r3* (398 bp). (PDF 249 kb)

Additional file 2: List of putative *cis*-acting regulatory elements present in the *StAN1* promoter. Promoter analysis was performed using New PLACE database. (PDF 51 kb)

Additional file 3: Schematic arrangement of primer pairs designed for qPCR analysis of *StAN1* gene. Arrows indicate primers: black – primer pair **a**. red – primer pair **b**. (PDF 67 kb)

## Abbreviations

AN1: Anthocyanin1
MBW: MYB, bHLH, WD40
