# Supplementary material for: Genetic control of anthocyanin pigmentation of potato tissues
Source: BMC Genet. 2019 Mar 18;20(Suppl 1):27. doi: 10.1186/s12863-019-0728-x (PMC6421638; doi:10.1186/s12863-019-0728-x)

Additional file 1.

PCR analysis of 36 samples of Russian potato varieties and hybrids.

r0 – *StAN1-r0* (296 bp), r1 – *StAN1-r1* (338 bp), r2 – *StAN1-r2* (377 bp), r3 – *StAN1-r3* (398 bp).

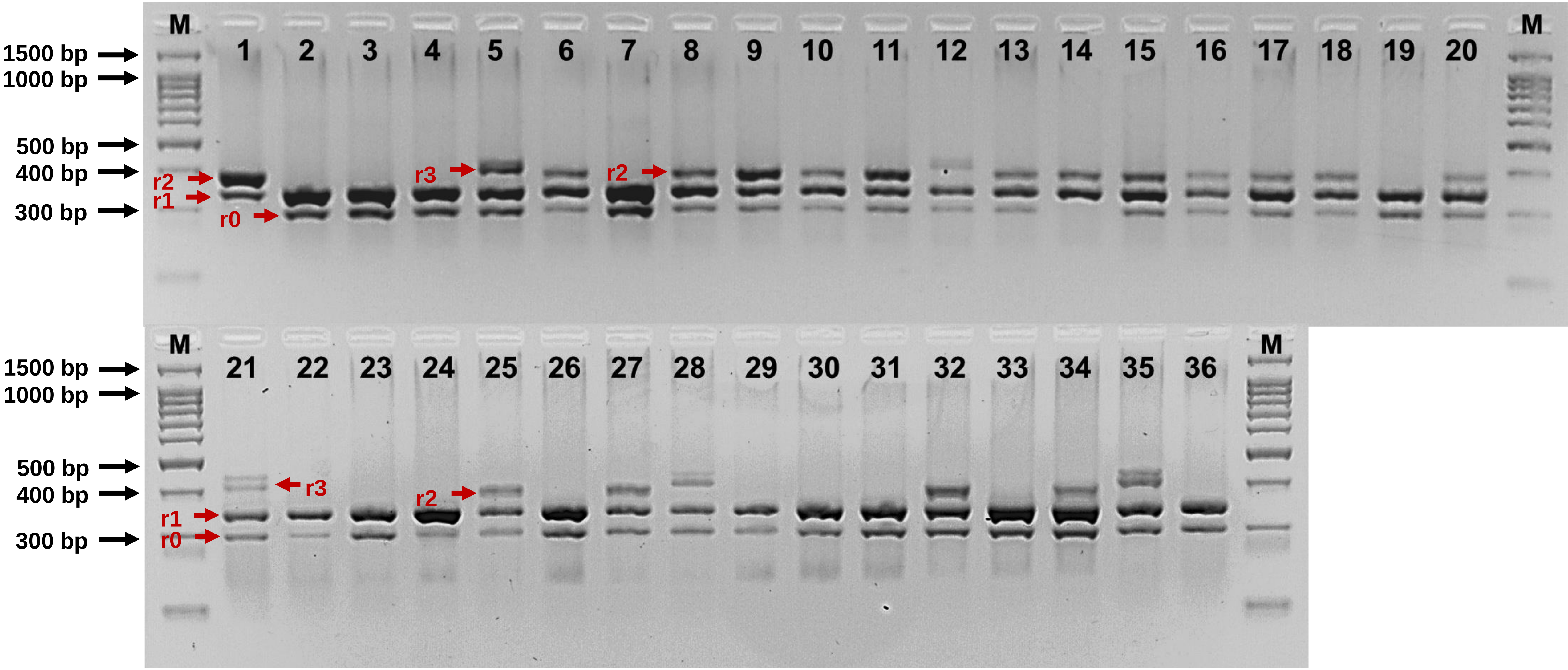

Supplement: Supplementary file 1 — PCR analysis of 36 samples of Russian potato varieties and hybrids. r0 – StAN1-r0 (296 bp), r1 – StAN1-r1 (338 bp), r2 – StAN1-r2 (377 bp), r3 – StAN1-r3 (398 bp). (PDF 249 kb) [file 12863_2019_728_MOESM1_ESM.pdf]
