# Supplementary material for: Genetic control of anthocyanin pigmentation of potato tissues
Source: BMC Genet. 2019 Mar 18;20(Suppl 1):27. doi: 10.1186/s12863-019-0728-x (PMC6421638; doi:10.1186/s12863-019-0728-x)
Supplement: Supplementary file 2 — List of putative cis-acting regulatory elements present in the StAN1 promoter. Promoter analysis was performed using New PLACE database. (PDF 51 kb) [file 12863_2019_728_MOESM2_ESM.pdf]

Additional file 2.

List of putative *cis*-acting regulatory elements present in the *StAN1* promoter. Promoter analysis was performed using New PLACE database.

**Genotype**			**Factor or Site Name**	**Signal Sequence**	**Function**
**Sequence from PGSC database**	**Zlatka, Yuna**	**Lina, Fioletovy, 710/10-5, 1014/1-8, 790/1-5**			
1	1	1	ROOTMOTIFTAPOX1	ATATT	Motif found both in promoters of rolD
1	1	1	LECPLEACS2	TAAAATAT	Core element in LeCp (tomato Cys protease) binding cis-element (from -715 to -675) in LeAcs2 gene
3	2	1	CACTFTPPCA1	YACT	Tetranucleotide (CACT) is a key component of Mem1 (mesophyll expression module 1); Y=T/C
1	2	1	DOFCOREZM	AAAG	Dof1 and Dof2 transcription factors are associated with expression of multiple genes involved in carbon metabolism in maize
1	1	0	PYRIMIDINEBOXOSRAMY1A	CCTTTT	Functional dissection of a sugar-repressed alpha-amylase gene (Ramy1A) promoter in rice embryos
1	1	0	OSE1ROOTNODULE	AAAGAT	One of the consensus sequence motifs of organ-specific elements (OSE) characteristic of the promoters activated in infected cells of root nodules
0	0	1	OSE2ROOTNODULE	CTCTT	One of the consensus sequence motifs of organ-specific elements (OSE) characteristic of the promoters activated in infected cells of root nodules
1	1	4	CAATBOX1	CAAT	"CAAT promoter consensus sequence" found in legA gene of pea
1	2	1	INRNTPSADB	YTCANTYY	"Inr (initiator)" elements found in the tobacco psaDb gene promoter without TATA boxes; Light-responsive transcription of psaDb depends on Inr, but not TATA box
1	1	0	GATABOX	GATA	"GATA box"; GATA motif in CaMV 35S promoter; Required for high level, light regulated, and tissue specific expression
1	1	4	ARR1AT	NGATT	"ARR1-binding element" found in Arabidopsis; ARR1 is a response regulator; N=G/A/C/T; AGATT
1	0	1	SORLREP3AT	TGTATATAT	one of "Sequences Over-Represented in Light-Repressed Promoters (SORLREPs) in Arabidopsis
1	1	0	CIACADIANLELHC	CAANNNNATC	Region necessary for circadian expression of tomato (L.e.) Lhc gene

2	0	2	ACGTATERD1	ACGT	ACGT sequence (from -155 to -152) required for etiolation-induced expression of erd1 (early responsive to dehydration) in Arabidopsis
0	2	2	MYCCONSENSUSAT	CANNTG	MYC recognition site found in the promoters of the dehydration-responsive gene rd22 and many other genes in Arabidopsis
1	0	0	IBOX	GATAAG	Conserved sequence upstream of light-regulated genes
0	1	1	GT1CONSENSUS	GRWAAW	Consensus GT-1 binding site in many light-regulated genes
0	1	0	MYBPZM	CCWACC	Core of consensus maize P (myb homolog) binding site; W=A/T
1	0	0	MYB1AT	WAACCA	MYB recognition site found in the promoters of the dehydration-responsive gene rd22 and many other genes in Arabidopsis; W=A/T
