# Supplementary material for: Genetic control of anthocyanin pigmentation of potato tissues
Source: BMC Genet. 2019 Mar 18;20(Suppl 1):27. doi: 10.1186/s12863-019-0728-x (PMC6421638; doi:10.1186/s12863-019-0728-x)

Additional file 3:

Schematic arrangement of primer pairs designed for qPCR analysis of *StAN1* gene.

Arrows indicate primers: black – primer pair **a**. red – primer pair **b**.

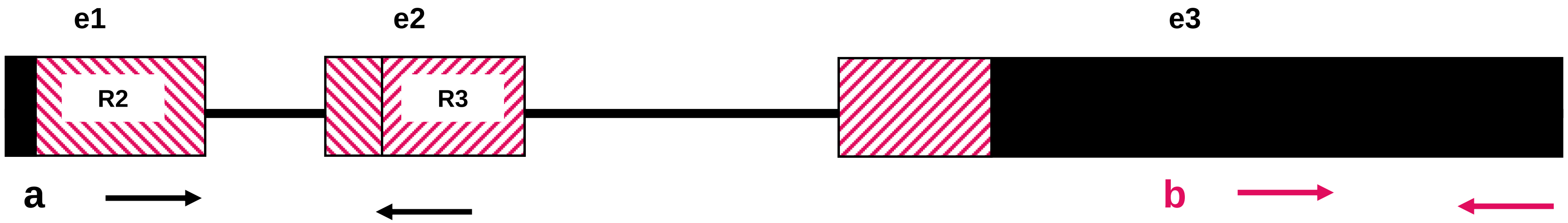

Supplement: Supplementary file 3 — Schematic arrangement of primer pairs designed for qPCR analysis of StAN1 gene. Arrows indicate primers: black – primer pair a. red – primer pair b. (PDF 67 kb) [file 12863_2019_728_MOESM3_ESM.pdf]
